# Detection, Differentiation and Quantification of Major Sugar Based Adulterants in Honey Using FTIR–ATR Spectroscopy Coupled with Chemometric Modeling

**DOI:** 10.3390/foods15183277

**Published:** 2026-09-17

**Authors:** Shanzeb Waseem, Muhammad Sohaib, Haroon Jamshaid Qazi, Mateen Abbas

**Affiliations:** 1 Department of Food Science and Human Nutrition, University of Veterinary and Animal Sciences, Lahore 54000, Pakistan; shanzebwaseem7@gmail.com (S.W.); haroon.jamshaid@uvas.edu.pk (H.J.Q.); 2 Institute of Microbiology, University of Veterinary and Animal Sciences, Lahore 54000, Pakistan; mateen.abbas@uvas.edu.pk

**Keywords:** honey adulteration, FTIR–ATR spectroscopy, chemometrics, food authentication, sugar adulterants

## Abstract

Honey is one of the most frequently adulterated food products owing to its higher commercial value and increased consumer demand. Its adulteration with inexpensive sugar syrups is a major challenge affecting honey authenticity and is resulting in decreased consumer confidence in the honey supply chain. The present study evaluated the capability of Fourier Transform Infrared Spectroscopy coupled with attenuated total reflectance (FTIR–ATR) and chemometric modeling to detect, classify, and quantify major sugar-based adulterants, namely fructose, glucose, invert sugar, jaggery syrup, maltose, and sucrose in honey samples. For the study, multifloral honey samples were experimentally adulterated with each adulterant @ 1, 5, 10, and 20% (*w*/*w*), and FTIR spectra were collected in the 650–4000 cm^−1^ region and carbohydrate fingerprint region (1500–750 cm^−1^) was used for multivariate analysis. Results indicated principal component analysis differentiated pure and adulterated honey samples with concentration-dependent clustering patterns while PLSR demonstrated excellent predictive performance. The calibration R^2^ values ranged from 0.9656 to 1.0000, whereas replicate-level hold-out evaluation gave R^2^ values of 0.9270–0.9999 with standard errors of prediction of 0.1342–2.0250%. Partial least squares discriminant analysis (PLS-DA) gave an overall sample-level classification accuracy of 92.0% in calibration and 88.0% in the hold-out dataset, with macro-averaged class accuracies of 97.71% and 96.57%, respectively. These findings demonstrate the potential of FTIR–ATR spectroscopy coupled with chemometric modeling as a rapid, non-destructive, reliable, and cost-effective alternative for routine honey authenticity testing, making it a valuable tool for quality control laboratories, regulatory agencies, and the honey industry.

## 1. Introduction

Honey is characterized by Codex Alimentarius Commission as “naturally occurring sweet substance that honeybees produce either from flower nectar or from different plant parts secretions, including plant-sucking insect’s excretions”. These substances are ultimately collected by bees which they convert into plant secretions by mixing with enzymes of their own, deposit and dehydrate followed by storage in honeycombs to mature and ripe [1]. Honey has been used since ancient times as a natural remedy with therapeutic benefits including anti-aging, anti-bacterial, and anti-inflammatory properties, boosting the immune system along with a hypoglycemic role as well as beneficial in wound healing, providing relief from sore throat, common cold, and treating conditions like skin infections, ulcers, etc. [2,3]. Honey is a complex natural food made up of around 200 different organic and inorganic components [4] with monosaccharides like glucose and fructose (≈80%) as major component, water (≈16%), ash (0.2%), some amino acids (<0.1%), along with phenolic compounds, minerals, organic acids, enzymes, and vitamins. The chemical composition and health benefits of honey are greatly influenced by its geographical and botanical sources [5,6].

Pakistan ranks 20^th^ among the world’s honey producers; its honey known for its unique sensory characteristics, quality attributes, and is considered a valuable agricultural commodity. Trade data from 2024 shows that Pakistan annually exports some 4000 tons of honey to Arab countries worth USD 23 million and its domestic consumption at approximately 11,000 tons annually [7,8]. The increasing commercial value of honey coupled with growing consumer demand has made honey highly susceptible to economically motivated adulteration. Although honey adulteration is a global concern as honey is among the top 10 most adulterated foods in Europe and the third most targeted food or commodity for fraud after milk and olive oil in the US. However, the honey-adulteration problem is particularly significant in Pakistan owing to weak regulatory oversight and particularly weak quality control system [5,9]. Several incidents have highlighted the seriousness of this issue. In 2017, the Punjab Food Authority confiscated approximately 30 tons of adulterated honey that had been mixed with industrial dyes [10]. Similarly, the Food Safety and Halal Food Authority of Pakistan seized 4000 L of counterfeit honey adulterated with sugars, chemicals, and wax in 2022 [11]. Furthermore, Kausar et al. [7] reported that 40% of raw honey samples collected from Punjab, Pakistan failed to comply with Punjab Pure Food Rules because of elevated hydroxymethylfurfural (HMF) levels, abnormal sugar composition, and higher moisture level than permitted. In the Pakistani context, honey authentication is further complicated by the diversity of botanical sources, seasonal variability, and differences in production and handling practices. Such variation can influence the chemical and spectral profiles of honey and should be considered when developing analytical models intended for broader application.

Natural honey, owing to its distinct flavor and nutritional benefits is more expensive than sugar sweeteners like corn and cane sugar. The increasing demand for quality honey with its limited supply has raised its market price, encouraging the addition of inexpensive sweeteners with similar chemical compositions to adulterate honey and deceive consumers regarding volume and quality [6,12]. In commercial settings, honey adulteration is generally categorized into three forms including direct addition, indirect, and blending of honey. Direct adulteration involves adding sugar syrups in certain ratios after production to increase the sweetness of honey and volume whereas indirect adulteration occurs when bees during peak nectar season are overfed with honey, industrial sugars, and chemicals to increase yield of honey from the beehives. Additionally, blending is another common practice in which premium higher quality honey is mixed with low quality pure and cheaper honey [2,13,14,15]. The literature reports that honey is commonly adulterated by adding glucose syrup, sucrose syrup (cane sugar), high fructose corn syrup along with beet and invert sugar syrups [14]. In Pakistan and India, jaggery and cane sugar are among frequently used adulterants [16]. The choice of substances used to adulterate honey is often influenced by three main factors like the region where honey is produced, potential economic gain from adulteration, and availability of sugars or sweeteners as per consumers’ taste and acceptability [2]. For the detection of honey adulteration several analytical techniques are used including thin layer chromatography (TLC), high-performance liquid chromatography (HPLC, gas chromatography), enzyme-linked immunosorbent assay (ELISA), and spectroscopy [17,18], (GC) [19] especially Raman [20], mid-infrared and NIR spectroscopy [21,22], along with nuclear magnetic resonance [23], and stable carbon isotopic ratio analysis [24], etc., that mainly depend on analysis, cost, specificity, and sensitivity of analysis.

These conventional analytical methods for routine honey adulteration testing are often limited in their application due to their time-consuming nature, destructive sample requirements and dependence on costly reagents and instrumentation. Therefore, development of alternative techniques with higher speed, affordability, and operational simplicity has become necessary [25]. The Fourier transform infrared attenuated total reflectance (FTIR–ATR) has emerged as a powerful vibrational spectroscopic technique that categorizes molecules by recognizing their unique molecular signatures as it is relatively fast, non-destructive, requires little or no sample preparation, and is a highly sensitive tool commonly used in food analysis and quality assurance [26]. At the same time, it has been recognized that the efficacy of FTIR–ATR spectroscopy can be further improved by integrating with chemometrics such as principal component analysis (PCA) and partial least squares (PLS) which are often used to extract useful patterns from complicated datasets [27]. Consequently, infrared spectroscopy combined with chemometric analysis (including both qualitative/quantitative) presents a highly effective solution for detecting adulteration in the honey supply chain.

Previously, FTIR–ATR spectroscopy integrated with chemometric analysis has been successfully applied to a variety of honey authentication purposes, including sugar profiling [28], determination of botanical and geographical origin [29,30], characterization of honey from different bee species [31], and detection of adulteration in conventional and stingless bee honeys [32,33,34]. In addition, several researchers have reported the successful identification and quantification of adulterants such as glucose, fructose, sucrose, corn syrup, high-fructose corn syrup, rice syrup, cane sugar syrup, beet sugar, and invert sugar using FTIR–ATR spectroscopy coupled with multivariate statistical approaches including PCA, PLSR, PLS-DA, LDA, CVA, and SIMCA [32,35,36,37,38,39,40]. Although these studies have demonstrated the strong potential of FTIR–ATR spectroscopy with chemometrics for honey authentication, most investigations have focused on a limited number of adulterants, individual syrup types, or specific honey varieties. The specific contribution of the present study is the evaluation of six individual sugar-based adulterants: fructose, glucose, sucrose, maltose, invert sugar, and jaggery syrup at four concentration levels (1, 5, 10, and 20% *w*/*w*) within a common FTIR–ATR/chemometric framework using honey from the Pakistani context. Addressing this gap, the present study was undertaken to investigate the potential of FTIR–ATR spectroscopy combined with chemometric techniques (PCA, PLSR, PLSDA) to detect, differentiate, and quantify major sugar adulterants in honey at different concentration levels, providing a rapid and reliable approach for honey authentication, and this study will provide baseline data for future authentication studies using spectroscopic techniques with special reference to the Pakistan honey supply chain.

## 2. Materials and Methods

The present study was conducted at the Food Safety Laboratory, Department of Food Science and Human Nutrition, in University of Veterinary and Animal Sciences Lahore, with objective to use Fourier transform infrared spectroscopy (FTIR) (Bruker ALPHA, Bruker Optics GmbH, Ettlingen, Germany) to detect and differentiate major adulterants in the pure versus adulterated honey samples.

### 2.1. Materials, Sampling and Experimental Design

For the study, a multifloral pure honey sample was collected from the Punjab Forest department Chichawatni, Punjab, Pakistan, a region recognized for its diverse floral vegetation and active apiculture practices. Honey was produced naturally by honeybees foraging on nectar from a variety of wild and cultivated floral sources present within forest ecosystem, thereby representing honey characteristics. Sample was collected as freshly harvested honey in clean, airtight amber glass containers to protect them from moisture absorption, light exposure, and external contamination during transportation and storage. The collected pure honey samples were stored under ambient temperature before they were subjected to any sort of preparation of pure versus adulterated honey samples for subsequent analytical measurements.

### 2.2. Preparation of Sugar Solutions and Honey Samples

For the sample preparation, fructose, glucose, sucrose, maltose, invert sugar, and jaggery as adulterants were prepared in this study. Among them, fructose syrup, glucose syrup, and malt syrup purchased from Bake house, Pakistan and Daraz, Pakistan whereas jaggery and cane sugar were purchased from Al Fatah Lahore, Pakistan. The malt syrup was used as a source of maltose. The commercially obtained syrup products were used according to their stated composition. Invert sugar syrup 70% (*w*/*w*) was prepared by combining 70 g sugar and 30 g distilled water with 0.15 g citric acid followed by boiling and mixing to get a sample of desired consistency. Thereafter, the mixture was cooled a bit, and 0.1 g sodium bicarbonate was added when the temperature reached 50 °C to neutralize the acidic mixture. Whereas, for the preparation of sucrose solution 70% (*w*/*w*), 70 g cane sugar was dissolved in 30 g distilled water under gentle heating with continuous stirring to attain a clear homogenous solution. Similarly, jaggery syrup was prepared by crushing jaggery blocks and mixing 70 g of jaggery in 30 g of water under gentle boiling with continuous stirring to obtain a homogenous 70% (*w*/*w*) jaggery syrup following the procedure of [41].

Afterwards, the adulterated honey samples were prepared by mixing pure honey directly with each one of six adulterant solutions including sucrose, glucose, fructose, maltose, jaggery, and invert sugar syrups. To enhance sensitivity at lower adulteration levels, samples were prepared in 5% increments up to 20%, as the identification of adulterants is typically more difficult at lower concentrations. Four levels of adulteration were prepared for each adulterant namely @1, 5, 10, and 20% (*w*/*w*) as reported in the experimental plan (Table 1). For example, a 10% adulterated sample was prepared by mixing 10 g of respective sugar solution with 90 g of pure honey with a total sum mass of honey and adulterants as 100 g. Similarly, other concentrations were prepared by proportionate mixing resulting in a total of 24 adulterated honey samples, with pure honey samples serving as a control group (100% pure). In adulteration, the honey proportion was 100–X%, where X is the percentage of adulterants solution. In addition to adulterated samples, a 100% adulterant solution (inverted sugar, sucrose solution, and jaggery solution) was also retained as a reference for spectral comparison of the selected adulterants. Because water-added or Brix-matched control formulations were not included, the contribution of differences in moisture or dilution to the observed spectral separation cannot be completely excluded. To ensure homogeneity, honey samples were mildly warmed (35–40 °C) prior to mixing to reduce the viscosity of the samples. The mixtures were then thoroughly homogenized using a glass rod until a uniform consistency was achieved. All sample mixtures were prepared and stored at room temperature overnight to ensure complete homogenization of their constituents prior to analysis.

### 2.3. Fourier-Transform Infrared Spectroscopy (FTIR) Analysis of Honey Samples

The mid-infrared (MIR) spectra of both pure and adulterated honey samples obtained using Bruker ALPHA, Bruker Optics GmbH, Ettlingen, Germany compact attenuated total reflectance Fourier Transform Infrared (ATR-FTIR) spectrometer, equipped with OPUS software version 7.5 and an ATR platinum diamond single-reflection accessory. Afterwards, the spectra were recorded over a wavenumber range of 650–4000 cm^−1^ at a nominal resolution of 4 cm^−1^. For each sample, 24 scans were collected, while 24 background scans were recorded before every measurement using a clean bare crystal surface. All spectra were recorded in absorbance mode with an approximate acquisition time of 30 s per scan. The liquid honey sample was placed directly on the diamond ATR crystal as a small drop for each spectral measurement, which does not require extensive sample preparation. To reduce the natural variation among samples, each sample was measured in triplicate to ensure reproducibility and reliability of spectral data. After each measurement, the crystal surface was thoroughly cleaned with tissue paper and then with ethanol to remove completely any residual sample avoiding carry-over contamination for successive analyses. The obtained spectra were analyzed using OPUS software to obtain absorbance values for further analysis.

### 2.4. Spectral Preprocessing

The FTIR–ATR dataset comprised 75 spectra obtained from 25 prepared honey formulations, with three spectral measurements collected for each formulation. Prior to chemometric analysis, the raw FTIR spectra were preprocessed before chemometric analysis to enhance spectral features and reduce baseline variations, which includes baseline correction using OPUS 7.5 software to remove additive effects. Subsequently, Savitzky–Golay second derivative (second polynomial order, 15-point window) and mean centering was applied using Chemoface v1.74 by following the procedure of [42]. Prior to multivariate analysis, this preprocessing step was carried out to resolve overlapped bands and reduce baseline effects.

### 2.5. Chemometric Analysis

The chemometric analysis was used to discriminate between pure and adulterated honey samples due to the ability of chemometric analysis to handle complex multivariate spectral data [34]. The room-temperature MIR FTIR–ATR spectra of both pure and adulterated honey samples were exported from the OPUS spectral acquisition software and directly imported into Chemoface software (version 1.74) for chemometric data analysis and model development. The preprocessed spectra pretreated with baseline, Savitzky–Golay second derivative (second polynomial order, 15-point window) and mean center were subsequently used for all chemometric analyses. For assessment of spectral differentiation, the fingerprint region (1500–750 cm^−1^), which contains the most informative carbohydrate-related absorption bands, was selected for chemometric modeling.

Qualitative analysis, including discrimination and classification of pure and adulterated honey samples, was performed using principal component analysis (PCA) and partial least squares discriminant analysis (PLS-DA). In addition, quantitative analysis for the prediction of adulterant concentration was carried out using partial least squares regression (PLSR). The chemometric model was developed by dividing the preprocessed spectral dataset into calibration and hold-out subsets using a stratified replicate-level split. For each of the 25 prepared formulations including pure and adulterated honey samples, two of the three measurement replicates were assigned to the calibration subset and the remaining replicate was assigned to the hold-out subset, resulting in 50 calibration spectra and 25 hold-out spectra. Because the three spectra originated from the same physical formulation, the hold-out subset does not represent fully independent external samples. Accordingly, the evaluation is referred to here as replicate-level hold-out evaluation and is interpreted as an assessment of model performance across measurement replicates of the prepared formulations rather than generalization to unseen honey formulations.

#### 2.5.1. Principal Component Analysis (PCA) for Exploratory Analysis

Principal component analysis (PCA) was applied as an unsupervised chemometric technique to explore patterns within the spectral dataset and visualize relationships among pure and adulterated honey samples [43]. PCA reduces the dimensionality of spectral data by transforming the original variables into a smaller number of principal components that capture the maximum variation present in the dataset. The analysis was performed on the preprocessed FTIR spectra to identify clustering behavior, sample distribution, and potential outliers. Score plots generated from the principal components were used to assess similarities and differences among the studied groups. The contribution of each principal component to the total explained variance was also evaluated. PCA served as a preliminary exploratory tool for investigating spectral variation associated with different adulterants and adulteration levels prior to supervised classification.

#### 2.5.2. Classification of Honey Using Partial Least Squares Discriminant Analysis (PLS-DA)

Partial least squares discriminant analysis (PLS-DA) was employed as a supervised classification method to discriminate between pure and adulterated honey samples. The calibration dataset containing known class labels was used to construct the classification model, while the replicate-level hold out validation dataset was used to evaluate its predictive performance [44]. The method identifies latent variables that maximize separation between predefined sample classes while minimizing within-class variation. The optimum number of latent variables was determined through cross-validation to achieve maximum classification performance and reduce the risk of overfitting. The samples may be correctly classified in their true class (true positive), wrongly assigned to another class (false positive), or not assigned to the correct class (false negative) thus reflecting the discriminatory power and predictive reliability of the developed model.

#### 2.5.3. Quantification of Adulterants Using Partial Least Squares Regression (PLSR)

The partial least squares regression (PLSR) was used as a supervised multivariate calibration method for the quantitative prediction of adulterant concentrations in honey samples using FTIR spectral data. PLSR establishes a relationship between spectral variables (X-matrix) and response variables, i.e., concentrations of individual adulterants (Y-variable), through latent variables (LVs). Following the qualitative classification of pure and adulterated honey samples using PCA and PLS-DA, PLSR was employed to predict adulterant concentrations in the samples [32]. Separate PLSR models were developed for each adulterant to improve prediction accuracy and minimize spectral interference among different adulterants. The calibration dataset was used to develop regression models, while the replicate-level hold-out subset was used to evaluate their performance across measurement replicates. To avoid overfitting, the optimum number of latent variables was selected through cross-validation based on the lowest root mean square error of cross-validation (RMSECV) and the highest coefficient of determination (R^2^). Model performance was further evaluated using the root mean square error of calibration (RMSEC) and root mean square error of prediction (RMSEP). Predicted concentrations were compared with actual values to assess the predictive accuracy of each model.

## 3. Results

### 3.1. Characterization of FTIR–ATR Spectrum of Pure Honey

**Figure 1:**
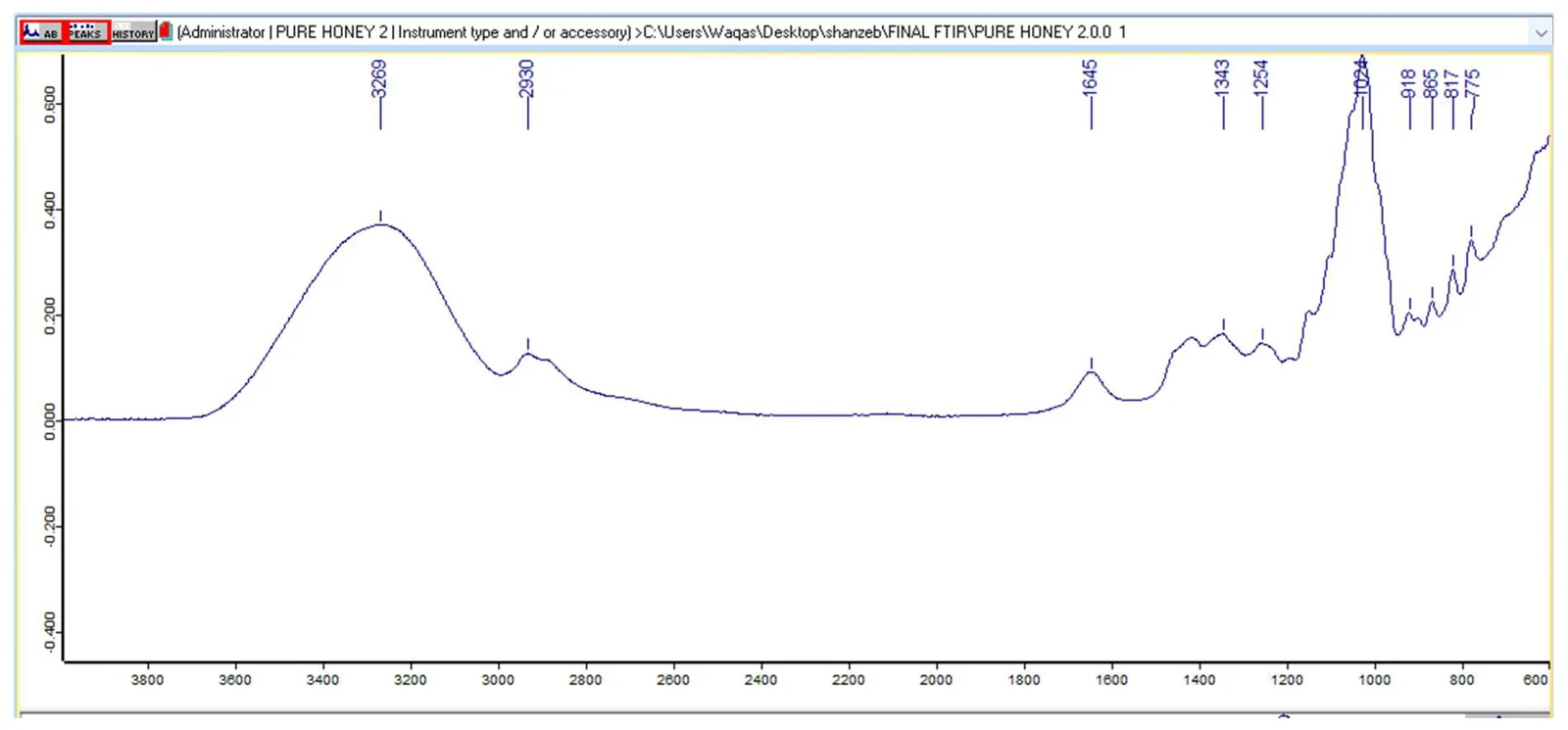
FTIR–ATR spectra of pure honey.

The FTIR–ATR spectrum of pure honey recorded in the mid-infrared region (4000–650 cm^−1^) is presented in Figure 1. The spectrum exhibited four major absorption zones with several characteristic absorption bands. A broad and intense absorption band was observed at around 3269 cm^−1^ primarily associated with O–H stretching, while another prominent band associated with O–H bending of water appeared at 1645 cm^−1^. A weak absorption band related to C–H stretching was detected at approximately 2930 cm^−1^.

The region between 1500 and 750 cm^−1^ contains overlapping carbohydrate-related C–O, C–C, and ring-vibration/deformation bands arising from the sugars that dominate honey composition. This region showed the most prominent spectral features of the spectrum. Absorption bands were observed at approximately 1343 and 1254 cm^−1^. The most intense absorption peak in the entire spectrum occurred at approximately 1024 cm^−1^. Further characteristic peaks were observed at approximately 918, 865, 817, and 775 cm^−1^.

The spectral profile showed distinct absorption features across the analyzed region, with the most pronounced spectral intensity occurring around 1024 cm^−1^ is therefore particularly informative for the carbohydrate-rich honey matrix and its adulterants. The region from 1500 to 750 cm^−1^ displayed multiple well-defined absorption bands, providing a characteristic fingerprint profile for the pure honey sample. Overall, the FTIR–ATR spectrum of pure honey showed a consistent spectral pattern across the analyzed mid-infrared region, with prominent absorption features at 3269, 1645, 1343, 1254, 1024, 918, 865, 817, and 775 cm^−1^.

### 3.2. Characterization of FTIR–ATR of Different Adulterants Solutions

**Figure 2:**
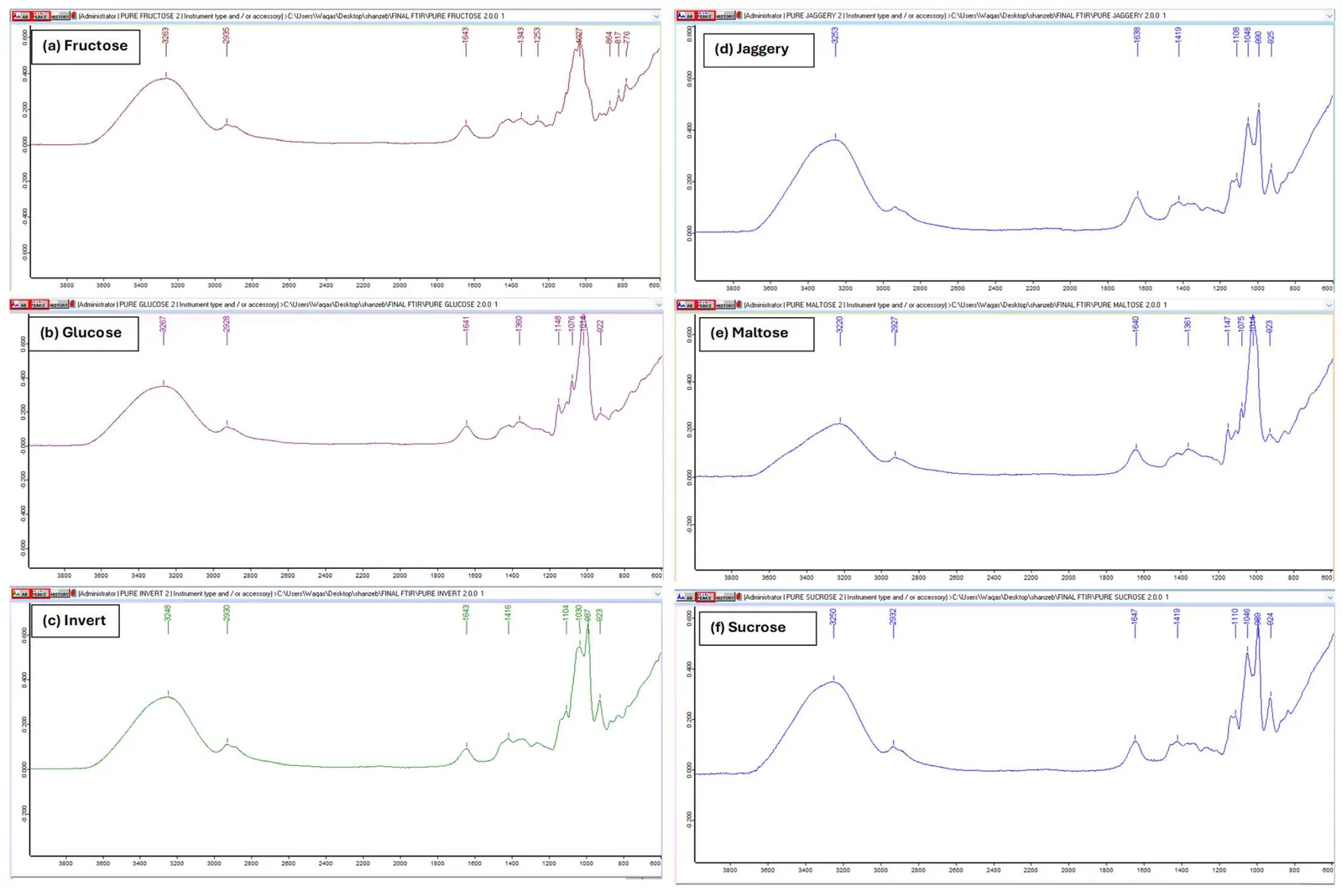
FTIR–ATR spectra of pure honey adulterants including fructose, glucose, invert sugar, jaggery, maltose, and sucrose.

The ATR–FTIR spectra of the different adulterants, including fructose, glucose, invert sugar, jaggery syrup, maltose, and sucrose, are presented in Figure 2. All adulterants exhibited characteristic absorption bands, with noticeable differences in peak intensity and slight shifts in band positions.

A broad and intense absorption band was observed in the region of approximately 3290–3260 cm^−1^ in all spectra. A weak absorption band was observed at approximately 2930–2920 cm^−1^, while another absorption band was observed around 1640–1645 cm^−1^. Additional absorption bands were observed in the region of 1450–1340 cm^−1^, particularly around 1415–1419 cm^−1^ and 1343–1345 cm^−1^. The most prominent differences among the adulterants were observed in the fingerprint region (1200–750 cm^−1^).

In Figure 2a, the fructose spectrum showed a strong absorption band around 1020–1025 cm^−1^. The glucose spectrum in Figure 2b also showed a strong absorption band around 1020–1025 cm^−1^. The invert sugar spectrum in Figure 2c exhibited characteristic absorption bands around 1104, 1021, and 923 cm^−1^. The maltose and sucrose spectra in Figure 2e,f exhibited sharper and more defined peaks in the 1100–1000 cm^−1^ region, particularly near 1110, 1045–1048, and 992–994 cm^−1^.

The jaggery syrup spectrum in Figure 2d also exhibited similar absorption bands, although broader peak features and variations in intensity were observed. Additional bands were observed in the region of 900–750 cm^−1^ at approximately 898, 867, 818, and 775 cm^−1^. Overall, the six adulterants showed distinct FTIR–ATR spectral profiles, particularly in the fingerprint region, with differences in peak positions, peak sharpness, and absorption intensity (Figure S1).

### 3.3. Characterization of FTIR–ATR of Honey Adulterated with Sugar Syrups

**Figure 3:**
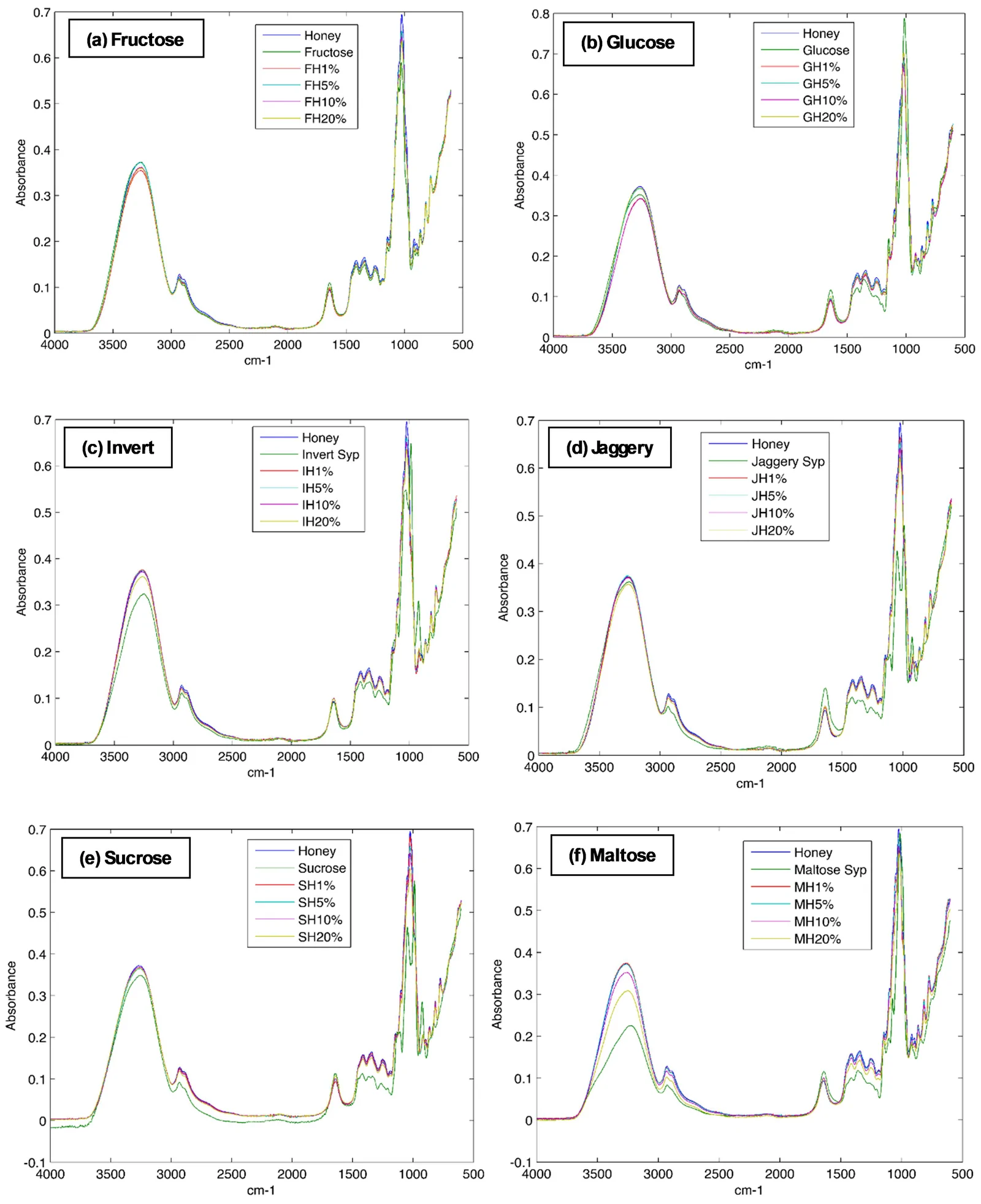
Comparative FTIR-ATR spectra of pure honey and honey samples adulterated with individual adulterants at different concentrations: (**a**) fructose; (**b**) glucose; (**c**) invert sugar; (**d**) jaggery syrup; (**e**) sucrose; and (**f**) maltose.

The ATR–FTIR spectra of honey samples adulterated with fructose, glucose, invert sugar, jaggery syrup, maltose, and sucrose at different concentration levels (1, 5, 10, and 20%) over the spectral range of 4000–650 cm^−1^ are presented in Figure 3. Overall, the adulterated samples retained a similar general spectral profile to pure honey, with characteristic absorption bands observed at similar wavenumbers.

The broad absorption band was observed in the range of 3280–3300 cm^−1^, while a weak band was observed around 2930 cm^−1^. All samples showed an absorption band around 1640–1645 cm^−1^. The major spectral variations were observed in the fingerprint region between 1500 and 750 cm^−1^, where absorption bands occurred at approximately 1340, 1250, 1148–1050, 1020–1030, 918, 867, 817, and 775 cm^−1^. With increasing adulteration levels from 1 to 20%, a systematic increase in absorbance intensity was observed, particularly in the 1100–1000 cm^−1^ region.

For fructose-adulterated samples in Figure 3a, prominent enhancement was observed near 1026 cm^−1^ and in the regions around 876 and 776 cm^−1^. Glucose-adulterated samples in Figure 3b exhibited increased intensity around 1021 cm^−1^, together with bands around 918 and 867 cm^−1^. Invert sugar-adulterated samples in Figure 3c showed an increase in absorption intensity around 1050 cm^−1^. For maltose-adulterated samples, clear increases in intensity were observed around 1147, 1078, and 1024 cm^−1^. Sucrose-adulterated samples showed distinct changes in the regions of 1100–1000 and 920–770 cm^−1^. The jaggery-adulterated samples in Figure 3d also showed systematic enhancement in absorption intensity, particularly around 1020–1030 cm^−1^. The spectra of the adulterated samples showed a gradual shift toward the spectral profiles of their respective pure adulterants with increasing adulteration levels, particularly at 10 and 20%. Samples containing 20% adulterant exhibited the greatest similarity to the corresponding pure adulterant spectra. Overall, the differences between pure and adulterated honey were mainly observed as changes in absorbance intensity and spectral distribution at selected characteristic bands, while the characteristic absorption bands remained at similar wavenumbers. The differences were more evident with increasing adulteration concentration, particularly within the fingerprint region.

### 3.4. Chemometric Analysis

#### 3.4.1. Principal Component Analysis (PCA) of Pure and Adulterated Honey Samples

**Figure 4:**
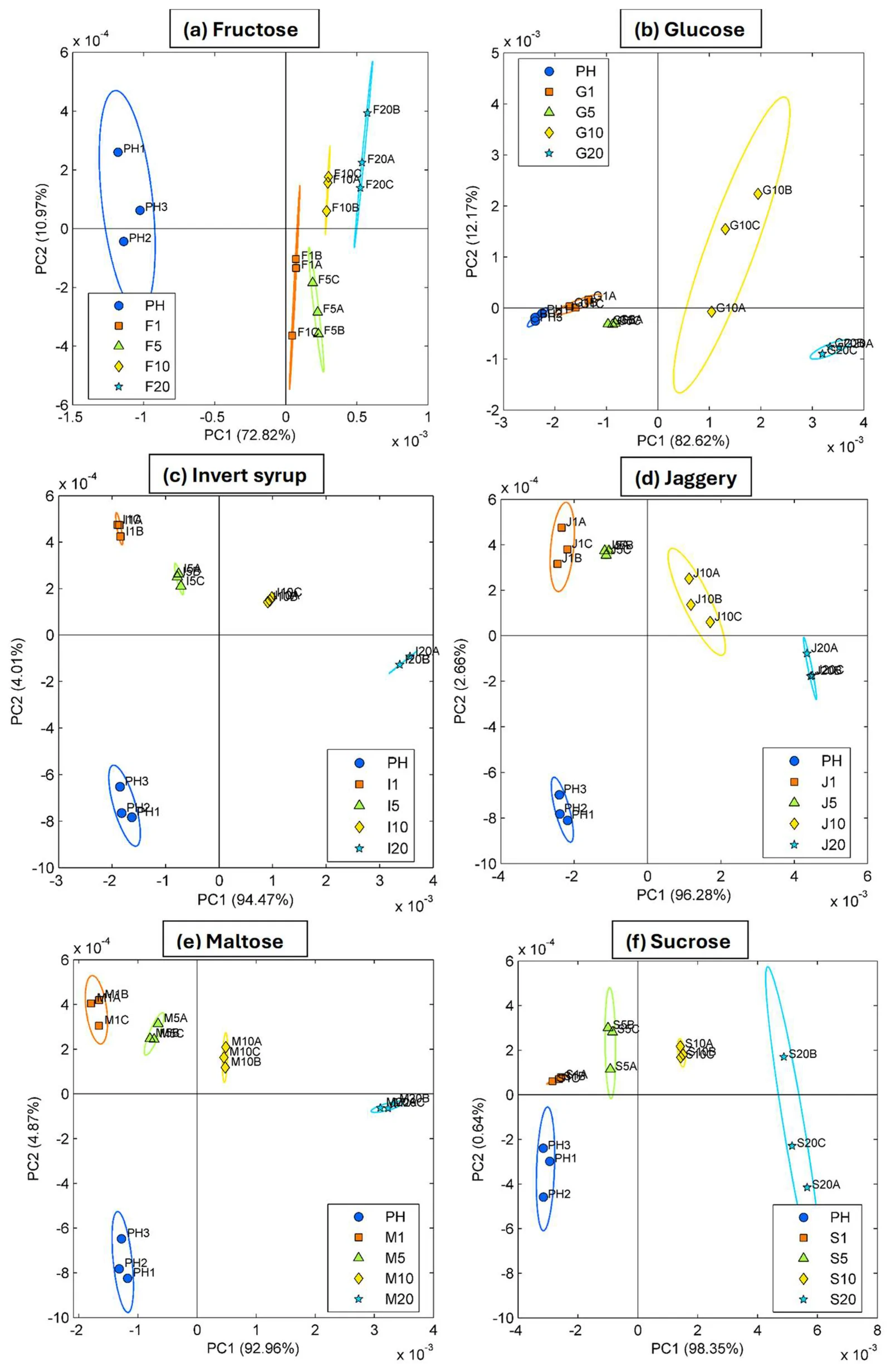
PCA score plots of different sugar syrups adulteration in honey.

The pre-processed FTIR–ATR spectral data were analyzed using principal component analysis (PCA) to assess the discrimination between pure honey and samples adulterated with different types of sugars at various concentrations. The PCA score plots based on the first two principal components (PC1 and PC2) are presented in Figure 4.

PC1 explained the highest proportion of the variance in all datasets, ranging from approximately 72% to 98%, while PC2 explained a lower but meaningful proportion of the variance. The PCA score plots showed clustering and separation between pure honey (PH) and adulterated samples. The pure honey samples formed compact and well-defined clusters. The adulterated samples were distributed separately from the pure honey clusters, with separation observed mainly along the PC1 axis. Samples with lower adulteration levels (1% and 5%) were positioned closer to the pure honey clusters, whereas samples containing 10% and 20% adulterant showed greater separation along PC1.

For the monosaccharide adulterants, fructose (Figure 4a) and glucose (Figure 4b) showed a gradual shift away from the pure honey cluster with increasing adulteration concentration. The samples containing 20% adulterant showed the greatest separation and were positioned furthest from the pure honey cluster. Invert sugar-adulterated samples (Figure 4c) showed a similar distribution pattern, with samples separated according to concentration level. For maltose and sucrose (Figure 4e,f), separation along PC1 was more pronounced, with distinct clustering according to concentration level. The variance explained in these models was greater than approximately 90%.

Jaggery-adulterated samples (Figure 4d) also showed clear clustering, although the clusters were comparatively broader. The separation between samples increased with increasing adulteration concentration. Overall, the PCA score plots showed separation between pure and adulterated honey samples and demonstrated concentration-dependent distribution patterns for the different adulterants.

#### 3.4.2. Determination of Adulterants Level in Honey Using Partial Least Squares Regression (PLSR)

Partial Least Squares Regression (PLSR) was applied to quantify the level of adulteration in honey samples using pre-processed FTIR–ATR spectral data. Independent PLSR models were developed for each adulterant to model concentration-dependent spectral variations. For each model, a total of fifteen spectra were analyzed and systematically divided into calibration (*n* = 10) and replicate level hold out validation (*n* = 5) sets. The calibration results are presented in Table 2.

The calibration models demonstrated coefficients of determination (R^2^-cal) ranging from 0.9656 to 1.0000. Invert sugar and sucrose exhibited the highest calibration performance, with R^2^-cal values of 1.0000 and SEC values of 0.0409% and 0.0139%, respectively. Glucose showed an R^2^-cal of 0.9951 with an SEC of 0.5095%, while maltose and jaggery showed R^2^-cal values of 0.9994 and 0.9993, with SEC values of 0.1754% and 0.1983%, respectively. The fructose model showed an R^2^-cal of 0.9656 and an SEC of 1.3539%.

The prediction performance of the models based on the separate hold out validation samples is also presented in Table 2. The R^2^-pred values ranged from 0.9270 to 0.9999, with SEP values ranging from 0.1342% to 2.0250%. Maltose showed the highest prediction performance, with an R^2^-pred of 0.9999 and SEP of 0.1342%, followed by invert sugar (R^2^-pred = 0.9990; SEP = 0.2525%) and sucrose (R^2^-pred = 0.9976; SEP = 0.4715%). Glucose showed an R^2^-pred of 0.9968 and SEP of 0.6108%, while jaggery showed an R^2^-pred of 0.9855 and SEP of 1.0299%. Fructose showed the lowest R^2^-pred value (0.9270) and the highest SEP value (2.0250%) among the tested adulterants.

**Figure 5:**
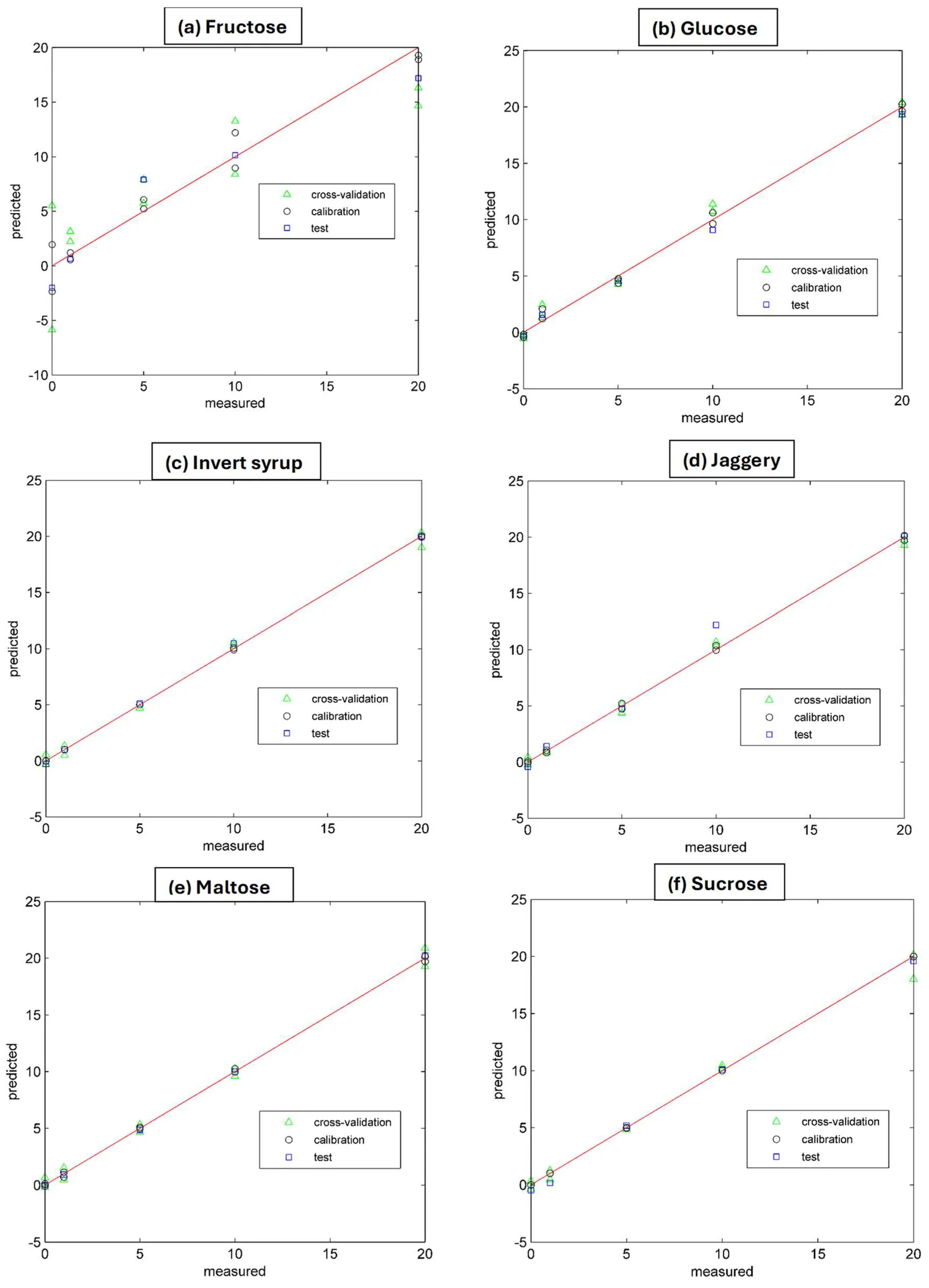
Predicted versus measured score plots for (**a**) fructose, (**b**) glucose, (**c**) invert, (**d**) jaggery, (**e**) maltose, and (**f**) sucrose syrup adulteration in honey via PLS method.

The regression plots of predicted versus measured values are presented in Figure 5. Most of the samples were distributed closely around the ideal prediction line. The maltose, sucrose, and invert sugar models showed close agreement between measured and predicted values, whereas comparatively greater variation was observed for the fructose and jaggery models. The number of latent variables used in the PLSR models ranged from two to six depending on the adulterant.

#### 3.4.3. Classification of Pure and Adulterated Samples Using Partial Least Squares Discriminant Analysis (PLS-DA)

Partial Least Squares Discriminant Analysis (PLS-DA) was used to classify pure honey and adulterated samples using pre-processed FTIR–ATR spectral data. The model was developed using 50 calibration samples and tested using a replicate level hold-out validation set of 25 samples. The classification performance was evaluated using the score plots and confusion matrices.

The class-wise results showed good classification performance for the investigated sample classes (Table 3). Pure honey, glucose, and sucrose showed 100% sensitivity, specificity, and class-wise accuracy in both the calibration and replicate level hold-out validation datasets. Fructose showed 100% sensitivity in both datasets, with a class-wise accuracy of 92.00% and specificity of 90.48%. Invert sugar showed a sensitivity of 87.50% and class-wise accuracy of 98.00% in the calibration dataset, which decreased to 75.00% sensitivity and 92.00% accuracy in the hold-out validation dataset. Similarly, jaggery showed 87.50% sensitivity and 98.00% accuracy during calibration, while the replicate level hold-out validation dataset showed 75.00% sensitivity and 96.00% accuracy. Maltose showed 75.00% sensitivity and 96.00% accuracy in calibration and 75.00% sensitivity and 96.00% accuracy in hold-out validation. The corresponding macro-averaged class accuracies were 97.71% for calibration and 96.57% for the hold-out dataset. Mean sensitivity was 92.86% and 89.29%, while mean specificity was 98.64% and 97.96%, respectively. The macro-averaged precision values were 95.24% and 91.67%. These results should be interpreted as replicate-level hold-out performance because measurement replicates from the same prepared formulations were represented in both subsets (Table 4). The PLS-DA model correctly classified 46 of 50 calibration spectra and 22 of 25 hold-out spectra, corresponding to overall sample-level accuracies of 92.0% and 88.0%, respectively as shown in Table 5 and Table 6. 

**Table 3:** Class-wise classification performance of PLS-DA model for honey authentication.

Classes	Dataset	No. of Samples (*n*)	TP	FP	FN	Sensitivity (%)	Specificity (%)	Accuracy (%)
Pure Honey	Calibration	2	2	0	0	100	100	100
	Replicate-level hold-out Validation	1	1	0	0	100	100	100
Fructose	Calibration	8	8	4	0	100	90.48	92
	Replicate-level hold-out Validation	4	4	2	0	100	90.48	92
Glucose	Calibration	8	8	0	0	100	100	100
	Replicate-level hold-out Validation	4	4	0	0	100	100	100
Invert Sugar	Calibration	8	7	0	1	87.50	100	98
	Replicate-level hold-out Validation	4	3	1	1	75.00	95.24	92
Jaggery	Calibration	8	7	0	1	87.50	100	98
	Replicate-level hold-out Validation	4	3	0	1	75.00	100	96
Maltose	Calibration	8	6	0	2	75.00	100	96
	Replicate-level hold-out Validation	4	3	0	1	75.00	100	96
Sucrose	Calibration	8	8	0	0	100	100	100
	Replicate-level hold-out Validation	4	4	0	0	100	100	100

**Table 4:** Overall classification performance of PLS-DA model for honey authentication.

Parameters	Calibration	Replicate Level Hold-Out Validation
Number of samples (*n*)	50	25
Overall sample-level accuracy (%)	92.00	88.00
Macro-averaged class accuracy (%)	97.71	96.57
Mean Specificity (%)	98.64	97.96
Mean Sensitivity (%)	92.86	89.29
Precision (%)	95.24	91.67
F1-score	0.9320	0.8949
MCC	0.9245	0.8816

**Table 5:** Seven-class confusion matrix of the PLS-DA calibration dataset.

Actual\Predicted	Pure Honey	Fructose	Glucose	Invert Sugar	Jaggery	Maltose	Sucrose
Pure Honey	**2**	0	0	0	0	0	0
Fructose	0	**8**	0	0	0	0	0
Glucose	0	0	**8**	0	0	0	0
Invert Sugar	0	1	0	**7**	0	0	0
Jaggery	0	1	0	0	**7**	0	0
Maltose	0	2	0	0	0	**6**	0
Sucrose	0	0	0	0	0	0	**8**

Bold values indicate correctly classified samples (diagonal elements), whereas non-bold values indicate misclassified samples.

**Table 6:** Seven-class confusion matrix for the replicate-level hold-out validation datasets.

Actual\Predicted	Pure	Fructose	Glucose	Invert	Jaggery	Maltose	Sucrose
Pure	**1**	0	0	0	0	0	0
Fructose	0	**4**	0	0	0	0	0
Glucose	0	0	**4**	0	0	0	0
Invert	0	**1**	0	**3**	0	0	0
Jaggery	0	0	0	**1**	**3**	0	0
Maltose	0	**1**	0	0	0	**3**	0
Sucrose	0	0	0	0	0	0	**4**

Bold = correctly classified observations.

**Figure 6:**
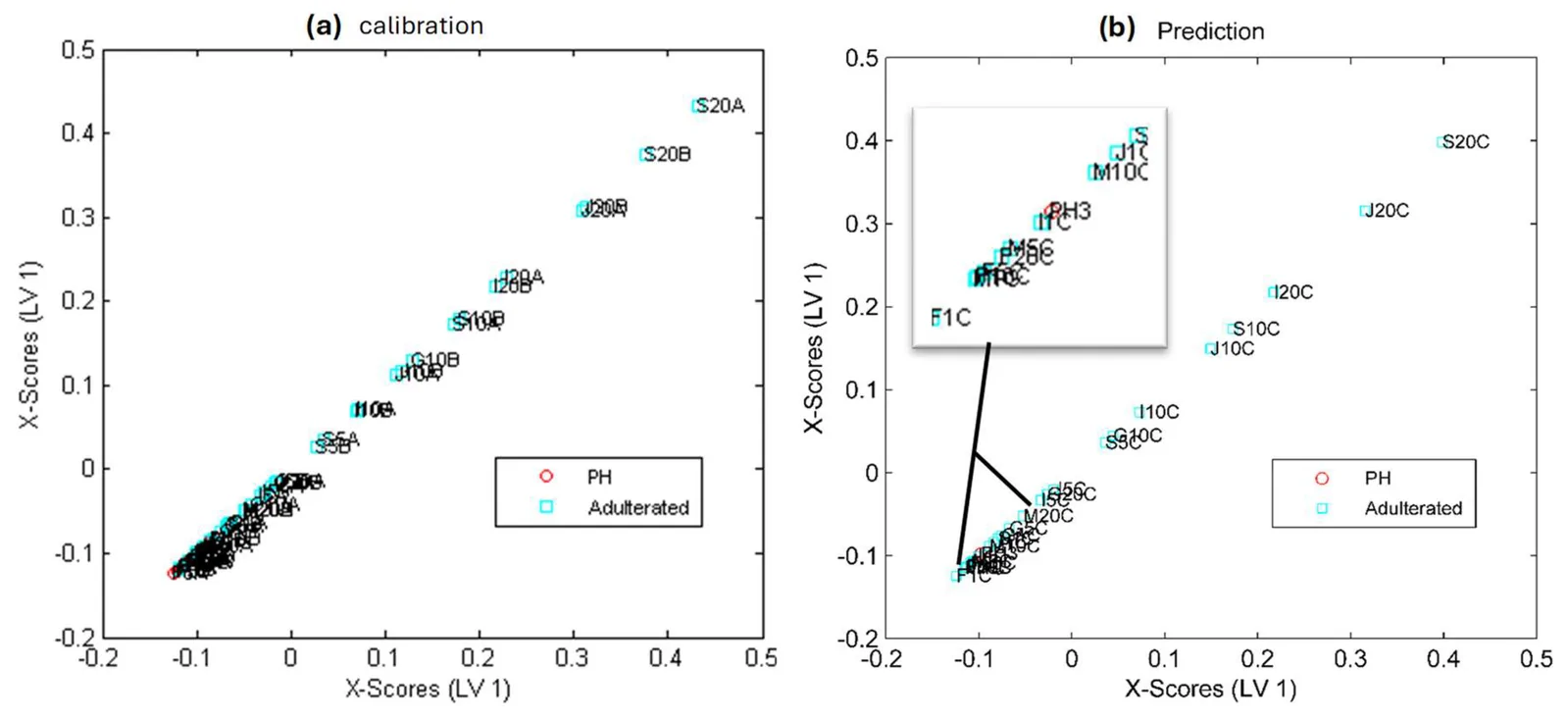
PLS-DA (**a**) Calibration and (**b**) Prediction score plot.

The PLS-DA calibration and prediction score plots shown in Figure 6a,b demonstrated separation between pure and adulterated samples, mainly along the first latent variable (LV1). A concentration-dependent distribution was observed as the adulteration level increased from 1% to 5%, 10%, and 20%. Samples containing 1% and 5% adulterant showed weaker separation and greater similarity to the pure honey cluster than samples containing 10% and 20% adulterant. Although the 1% and 5% samples were distinguishable within the present dataset, a formal limit of detection or limit of quantification was not established. The Figure 6 showed separation between pure and adulterated samples, with greater separation generally observed at higher adulteration levels. The enlarged view in Figure 6b further showed the distribution of samples at the lower adulteration levels.

## 4. Discussion

The present study demonstrated the applicability of FTIR–ATR spectroscopy combined with chemometric analysis for the detection, differentiation, classification, and quantification of major sugar adulterants in honey. The FTIR spectra of pure honey, individual adulterants, and adulterated honey samples showed that the spectral analysis performed over the mid-infrared region (4000–650 cm^−1^) revealed the most significant discriminatory information concentrated within the fingerprint region (1500–750 cm^−1^), which was therefore selected for chemometric modeling. This observation is in strong agreement with previous studies, where optimal discrimination of honey adulteration has been reported within similar spectral regions. Damto, Zewdu and Birhanu [45] and Sivakesava and Irudayaraj [37] highlighted that the spectral interval between 1800 and 750 cm^−1^ was most effective for differentiating pure honey from samples adulterated with simple sugars such as fructose, sucrose, and glucose. According to Gallardo-Velázquez, Osorio-Revilla, Zuñiga-de Loa, and Rivera-Espinoza [32] the regions of 1500 and 750 cm^−1^ are of significant importance for honey and similar products, as they are the regions for absorption of primary monosaccharides and disaccharides. Likewise, Limm, Karunathilaka, and Mossoba [38] noted that that the fingerprint spectral region extending from 1500 to 800 cm^−1^ is largely shaped by characteristic absorption bands associated with the major sugar constituents of honey, including monosaccharides and disaccharides, thus supporting the importance of this spectral region for honey authentication.

The spectral profiles of the adulterated honey and authentic honey samples were highly overlapped, and no new peaks were observed on adulteration. Instead, the differences were mainly shown in the variations of the peak intensities in the fingerprint region, particularly in the regions of 1200–1000 cm^−1^ and 950–750 cm^−1^. This aligns with findings of [12,29], who observed that authentic and adulterated honey exhibit similar spectral features and the differentiation is based on the changes in intensity and not on the appearance of new functional groups. This shows that sugar adulteration does not introduce new chemical functionalities but changes the relative concentration of existing carbohydrate components, making reliable visual differentiation and quantitative assessment challenging, when based on individual spectral bands alone.

The PCA was applied to the present data set and was able to capture the subtle spectral variations among authentic and adulterated samples of honey. The first principal component accounted for a significant portion of the total variance (about 72–98%). The PCA score plots clearly clustered the samples of pure honey, which were separated clearly from the adulterated samples, confirming high discrimination potential of the selected fingerprint region (1500–750 cm^−1^). A concentration-dependent distribution pattern was also observed with samples with higher adulteration levels (10–20%) deviating more from pure honey than those with lower concentrations (1–5%). This progressive shift implies that the spectral profile is progressively modified by increasing adulterant levels as a result of changes in carbohydrate composition. Similar clustering behavior and high variance explanation have been reported in previous studies where PCA differentiated honey samples based on compositional variations in sugars [30,46].

In addition to the separation of classes, this study demonstrated differences in the discrimination of individual adulterants. Disaccharide adulterants, for example sucrose and maltose, exhibited better separation than monosaccharide adulterants, for example glucose and fructose. This is due to their more complex molecular structures and the presence of glycosidic linkages leading to different vibrational characteristics in the fingerprint region. Consistent findings were observed by Gok, Severcan, Goormaghtigh, Kandemir, and Severcan [29], who pointed out that carbohydrate structure differences have a large effect on FTIR spectral patterns and improve classification performance. The relatively lower separation of monosaccharides can be attributed to their natural existence in honey leading to higher spectral overlaps with the authentic samples.

The PLSR models showed strong fit and replicate-level hold-out performance within the controlled experimental dataset, with calibration R^2^-cal values of 0.9656–1.0000 and hold-out R^2^-pred values of 0.9270–0.9999. These results are consistent with earlier studies where FTIR-based PLS models generated high prediction accuracy with R^2^ values mostly exceeding 0.90 and low prediction errors [33,36]. Some recent studies have reported a higher predictive performance (R^2^ ≈ 0.99), confirming the reliability of FTIR coupled with chemometric techniques for the quantification of honey adulteration [30,40]. Similarly, Gallardo-Velázquez, Osorio-Revilla, Zuñiga-de Loa, and Rivera-Espinoza [32] demonstrated the reliable prediction of syrup adulterants with low values of SEP. The lower prediction accuracy of fructose in this study may be due to the fact that fructose is a natural component of honey and therefore has overlapping spectra with the endogenous sugars. These results support the feasibility of FTIR–ATR spectra for quantitative modeling of the tested adulteration formulations, but the current replicate level hold-out validation design does not establish robustness across unseen honey formulations or commercial samples.

In the present study, PLS-DA provided good classification performance for the defined sample classes under the replicate-level hold-out design. Unsupervised methods like PCA are useful for visualizing clustering patterns but do not give an exact classification especially in case of low levels of adulteration [41,45]. The study reached high classification accuracy for calibration (97.71%) and replicate level hold-out validation (96.57%) datasets, and pure honey samples were identified correctly in all the cases. Similar results have been reported in the literature where FTIR-based multivariate models achieved classification accuracy between 90 and 100% based on the complexity of samples and adulterant type [31,47]. Ciursă, Pauliuc, Dranca, Ropciuc, and Oroian [12] also reported generally between 74% and above 90% classification accuracies for the PLS-DA model, depending on the pre-processing techniques and model conditions. The minor misclassifications of the invert sugar, jaggery, and maltose samples in the study could be due to the similar carbohydrate compositions of these samples resulting in overlapping spectral signatures. Ciursă, Pauliuc, Dranca, Ropciuc, and Oroian [12] also reported similar challenges and emphasized the difficulty of differentiating chemically related adulterants by using spectral data alone.

One of the major outcomes of this work is the spectral variation depending on the concentration. Systematic variation in the absorbance intensity and chemometric score distributions are observed with increasing levels of adulterant. This behavior is in agreement with the high sensitivity of FTIR spectroscopy to gradual compositional changes in honey, allowing qualitative discrimination as well as prediction in a quantitative way. The same tendencies have been reported in previous works, where the spectral intensity changes were directly related to the quantity of the adulterant [30,32], which gives more support to the applicability of this technique.

The developed models are performing well but there are some limitations which need to be discussed. The high compositional similarity of the sugars present in natural honey and those of the added adulterants results in overlapping absorption bands which make the direct interpretation of the spectra difficult, without the aid of chemometric analysis. Such phenomena are described in the literature where it is stated that the absorption bands of honey and sugar syrups are similar and sophisticated statistical tools are required to differentiate them [12,48]. Further, the 1% and 5% adulteration levels showed weaker separation and greater similarity to pure honey than the higher concentrations. Similar results have been reported by Limm, Karunathilaka, and Mossoba [38], who reported the need for more advanced or highly sensitive modeling approaches for reliable detection of low-level adulteration. Although these tested levels were distinguishable within the present experimental dataset, the study did not establish a formal limit of detection or limit of quantification. Moreover, the differences in botanical and geographical origin may influence the composition and spectral characteristics of honey and may influence the generalizability of the model as mentioned earlier by [35].

The present study has some limitations. First, the experimental design was based on a single honey source and a limited number of independently prepared formulations. Second, three measurement replicates were collected from each formulation and distributed between calibration and hold-out subsets; therefore, the present validation evaluates replicate-level performance rather than prediction of completely unseen formulations. Thirdly, only individual adulterants were evaluated within a common analytical framework; mixtures containing multiple adulterants were not investigated. Finally, the influence of botanical origin, geographic origin, harvest batch, and commercial processing was not systematically evaluated. These factors should be addressed in future validation studies before wider regulatory or industrial application.

Overall, the results of the present study are in good agreement with the literature and demonstrate that FTIR–ATR spectroscopy along with chemometric analysis is a fast, non-destructive, and reliable method for the differentiation, detection, and quantification of major honey adulterants making it a promising laboratory screening approach for controlled sugar adulteration in honey. The rapid acquisition and minimal sample preparation of ATR–FTIR make the technique attractive for further development, but independent validation using diverse honey sources, commercial samples, and appropriately grouped calibration and validation datasets is required before routine regulatory or industrial implementation can be recommended.

## 5. Conclusions

In summary, FTIR–ATR spectroscopy coupled with chemometric analysis proved to be a rapid, non-destructive, and reliable approach for the detection, differentiation, classification, and quantification of major sugar adulterants in honey. The fingerprint region (1500–750 cm^−1^) provided the most relevant spectral information for honey authentication, while PCA, PLSR, and PLS-DA successfully discriminated pure honey from samples adulterated with fructose, glucose, sucrose, maltose, invert sugar, and jaggery syrup. The developed models demonstrated excellent classification and predictive performance within the replicate-level hold-out evaluation, highlighting the potential of this integrated analytical approach for routine honey quality assessment and fraud detection. Although discrimination at very low adulteration levels remains challenging due to the natural sugar composition of honey, the results confirm the suitability of FTIR–ATR spectroscopy as an efficient tool for honey authentication. However, because the present dataset was derived from a single honey source and replicate spectra from the same formulations were distributed across calibration and hold-out subsets, further validation with independently prepared and commercially sourced samples is necessary before routine regulatory or industrial implementation. Future research should focus on validating the developed models using a wider range of honey varieties, geographical origins, and adulteration scenarios to further enhance their robustness and practical applicability.

## Figures and Tables

**Table 1:** Study plan for preparation of pure and adulterated honey samples.

Treatments	Description
T_0_	100% Pure Honey
T_1_	99% Pure Honey + 1% Sucrose Syrup
T_2_	95% Pure Honey + 5% Sucrose Syrup
T_3_	90% Pure Honey + 10% Sucrose Syrup
T_4_	80% Pure Honey + 20% Sucrose Syrup
T_5_	99% Pure Honey + 1% Glucose Syrup
T_6_	95% Pure Honey + 5% Glucose Syrup
T_7_	90% Pure Honey + 10% Glucose Syrup
T_8_	80% Pure Honey + 20% Glucose Syrup
T_9_	99% Pure Honey + 1% Fructose Syrup
T_10_	95% Pure Honey + 5% Fructose Syrup
T_11_	90% Pure Honey + 10% Fructose Syrup
T_12_	80% Pure Honey + 20% Fructose Syrup
T_13_	99% Pure Honey + 1% Maltose Syrup
T_14_	95% Pure Honey + 5% Maltose Syrup
T_15_	90% Pure Honey + 10% Maltose Syrup
T_16_	80% Pure Honey + 20% Maltose Syrup
T_17_	99% Pure Honey + 1% Jaggery Syrup
T_18_	95% Pure Honey + 5% Jaggery Syrup
T_19_	90% Pure Honey + 10% Jaggery Syrup
T_20_	80% Pure Honey + 20% Jaggery Syrup
T_21_	99% Pure Honey + 1% Invert Sugar Syrup
T_22_	95% Pure Honey + 5% Invert Sugar Syrup
T_23_	90% Pure Honey + 10% Invert Sugar Syrup
T_24_	80% Pure Honey + 20% Invert Sugar Syrup

**Table 2:** Calibration and prediction parameters for fructose, glucose, invert sugar, jaggery, maltose, and sucrose adulteration in honey using PLS regression method.

Adulterated Samples	Calibration			Prediction	
	No of Factor	R^2^	SEC (%)	R^2^	SEP (%)
Fructose-Adulterated Honey	3	0.9656	1.3539	0.9270	2.0250
Glucose-Adulterated Honey	2	0.9951	0.5095	0.9968	0.6108
Invert-Adulterated Honey	4	1.0000	0.0409	0.9990	0.2525
Jaggery-Adulterated Honey	3	0.9993	0.1983	0.9855	1.0299
Maltose-Adulterated Honey	3	0.9994	0.1754	0.9999	0.1342
Sucrose-Adulterated Honey	6	1.0000	0.0139	0.9976	0.4715

## Data Availability

The processed spectral data, sample identifiers, and analytical results supporting the findings of this study are available from the corresponding author upon reasonable request. Additional information required to reproduce the chemometric analyses will be provided where appropriate.

## Supplementary Materials

The following supporting information can be downloaded at: https://www.mdpi.com/article/10.3390/foods15183277/s1, Figure S1: FTIR–ATR spectra of natural honey and the six adulterant solutions plotted on a common wavenumber axis with vertical offsets for direct visual comparison.

## Abbreviations

The following abbreviations are used in this manuscript:

FTIR–ATR	Fourier Transform Infrared Spectroscopy–Attenuated Total Reflectance
MIR	Mid Infrared Region
PCA	Principal Component Analysis
PLSR	Partial Least Square Regression
PLS-DA	Partial Least Square-Discriminant Analysis
R^2^	Coefficient of Determination
SEP	Standard Error of Prediction
SEC	Standard Error of Calibration
