# Supplementary material for: Detection, Differentiation and Quantification of Major Sugar Based Adulterants in Honey Using FTIR–ATR Spectroscopy Coupled with Chemometric Modeling
Source: Foods. 2026 Sep 17;15(18):3277. doi: 10.3390/foods15183277 (PMC13606338; doi:10.3390/foods15183277)
Supplement: Supplementary file 1 [file foods-15-03277-s001.zip › foods-4528126-supplementary.pdf]

Supplementary Material: The following supporting information includes **Figure S1.**

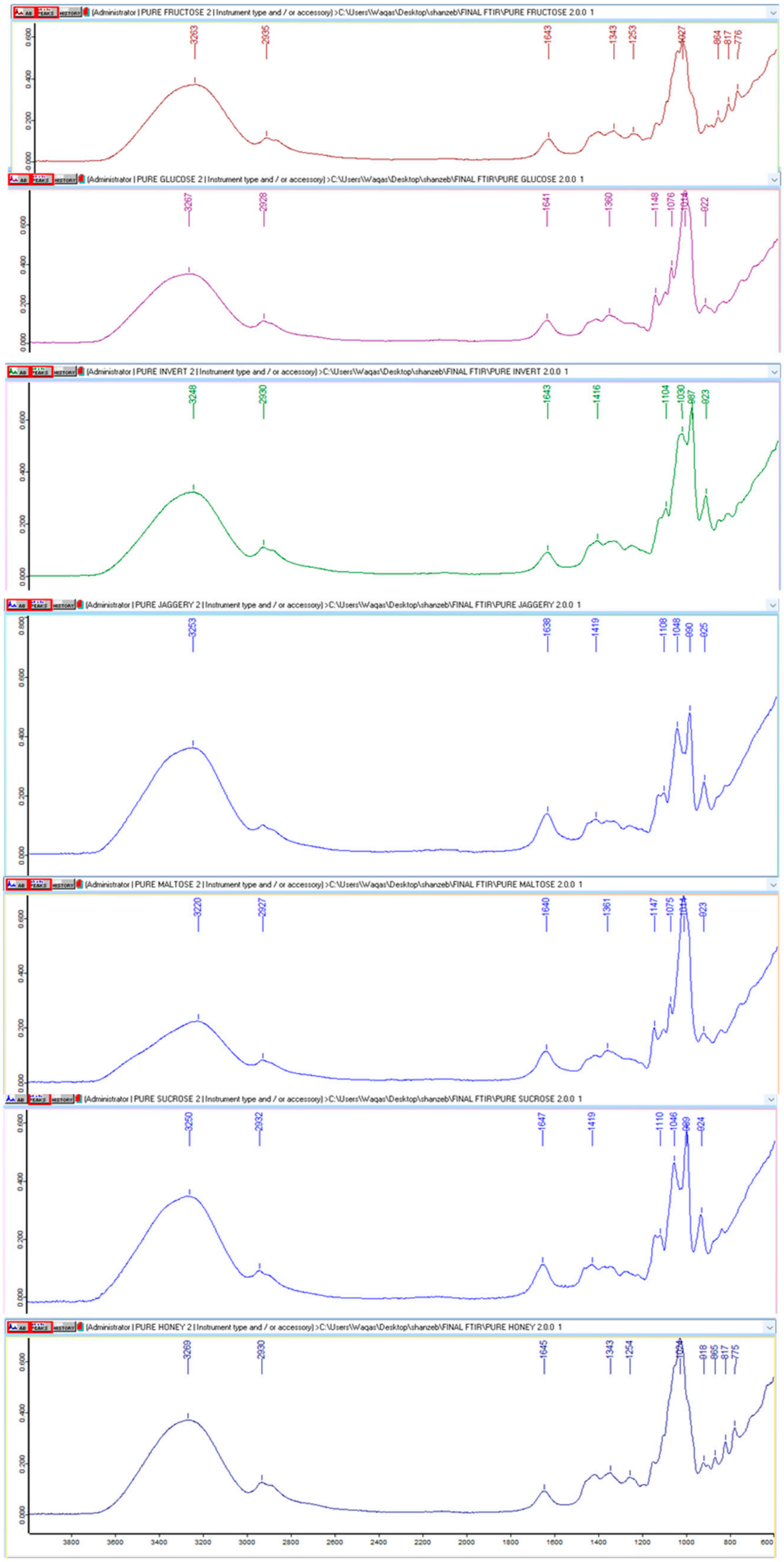

Figure S1: FTIR-ATR spectra of natural honey and the six adulterant solutions plotted on a common wavenumber axis with vertical offsets for direct visual comparison
